# *In-vitro* studies on the sensitivity pattern of *Plasmodium falciparum* to antimalarial drugs and local herbal extracts

**DOI:** 10.1186/1475-2875-11-S1-P72

**Published:** 2012-10-15

**Authors:** Grace Olasehinde, Olusola Ojurogbe, Obashola Fagade, Singh Ruchi, Adesola Ajayi

**Affiliations:** 1 Covenant University, Ota, Nigeria; 2 LAUTECH College of Medicine, Osogbo, Nigeria; 3 University of Ibadan, Nigeria; 4 NIMR, Delhi, India

## 

The resistance of human malaria parasites to antimalarial compounds has become of considerable concern, particularly in view of the shortage of novel classes of antimalarial drugs. One way to prevent resistance is by using new compounds that are not based on existing synthetic antimicrobial agents. Sensitivity of one hundred (100) *P. falciparum* isolates to chloroquine, quinine, amodiaquine, mefloquine, sulphadoxine/pyrimethamine, artemisinin, *Momordica charantia* (*Ejirin*,) *Diospyros monbuttensis* (*Eegun eja*) and *Morinda lucida* (*Oruwo*) was determined using the *in-vitro* microtest (Mark III) technique to determine the IC_50_ of the drugs. All the isolates tested were sensitive to Quinine, Mefloquine and Artesunate. Only 51% of the isolates were resistant to chloroquine, 13% to amodiaquine and 5% to sulphadoxine pyrimethamine respectively. Highest resistance to chloroquine (68.9%) was recorded among isolates from Yewa zone while highest resistance to amodiaquine (30%) was observed in Ijebu zone. Highest resistance to sulphadoxine and pyrimethamine was recorded in Yewa and Egba zones respectively. A significant positive correlation was observed between the responses to artemisinin and mefloquine (P=0.001), artemisinin and quinine (P=0.05), Quinine and mefloquine (P= 0.01). A significant negative correlation was observed between the responses to chloroquine and mefloquine (P=0.05). Highest antiplasmodial activity was obtained with the ethanolic extract of *Diospyros monbuttensis* (IC_50_ = 32 µg/ml) while the lowest was obtained from *Morinda lucida* (IC_50_ =250 µg/ml). Natural products isolated from plants used in traditional medicine, which have potent antiplasmodial action *in vitro*, represents potential sources of new antimalarial drugs.

